# Fruitomics: The Importance of Combining Sensory and Chemical Analyses in Assessing Cold Storage Responses of Six Peach (*Prunus persica* L. Batsch) Cultivars

**DOI:** 10.3390/foods11172554

**Published:** 2022-08-24

**Authors:** Antonella Muto, Sarah R. Christofides, Tiziana Maria Sirangelo, Lucia Bartella, Carsten Muller, Leonardo Di Donna, Innocenzo Muzzalupo, Leonardo Bruno, Antonio Ferrante, Adriana A. C. Chiappetta, Maria Beatrice Bitonti, Hilary J. Rogers, Natasha Damiana Spadafora

**Affiliations:** 1 Department of Biology, Ecology and Earth Sciences, University of Calabria, Via Ponte P. Bucci 6b, Arcavacata di Rende, 87036 Cosenza, Italy; 2 School of Biosciences, Cardiff University, Sir Martin Evans Building, Museum Avenue, Cardiff CF10 3AX, UK; 3 CREA—Council for Agricultural Research and Agricultural Economy Analysis, Genomics and Bioinformatics Research Center, 26836 Montanaso Lombardo, Italy; 4 Department of Chemistry and Chemical Technologies, University of Calabria, Via Ponte P. Bucci 12c, 87036 Rende, Italy; 5 CREA—Council for Agricultural Research and Agricultural Economy Analysis, Forestry and Wood Research Center, 87036 Rende, Italy; 6 Department of Agricultural and Environmental Science–Production, Landscape, Agroenergy, Università degli Studi di Milano, Via Celoria 2, 20133 Milan, Italy; 7 Markes International Ltd., 1000B Central Park, Western Avenue, Bridgend CF31 3RT, UK; 8 Department of Chemical, Pharmaceutical and Agricultural Sciences, University of Ferrara, 44121 Ferrara, Italy

**Keywords:** *Prunus persica*, phytochemical, post-harvest cold storage, sensorial analysis, volatile organic compounds

## Abstract

Cold storage is used to extend peach commercial life, but can affect quality. Quality changes are assessed through the content of nutritionally relevant compounds, aroma, physical characters and/or sensorially. Here, six peach and nectarine cultivars were sampled at commercial harvest and after 7 days of 1 °C storage. A trained panel was used to evaluate sensorial characters, while carotenoids, phenolics, vitamin C, total sugars, and qualitative traits including firmness, titrable acidity and soluble solid content were integrated with volatile organic compound (VOC) analysis previously reported. The different analyses reveal interesting patterns of correlation, and the six cultivars responded differently to cold storage. Sensory parameters were correlated with 64 VOCs and seven intrinsic characters. Acidity, firmness, and 10 VOCs were strongly negatively correlated with harmony and sweetness, but positively correlated with bitterness, astringency, and crunchiness. In contrast, Brix, b-carotene, and six VOCs were positively correlated with harmony and sweetness.

## 1. Introduction

Peach (*Prunus persica* L. Batsch) is an economically important crop, widely appreciated for its nutritional value and the flavour of its fruits. Currently, the major producers are China and several Mediterranean countries: Spain, followed by Italy, Turkey, and Greece [1]. The fruit is characterized by an outer skin (exocarp), a fleshy mesocarp, and a hard inner stone (endocarp) that contains the seeds. Peach and its variety nectarine have fruits with similar taste and appearance; the former have a silky, hairy skin, while the latter is smooth, differing only by a single gene [2]. Thousands of cultivars have been selected world-wide [3], showing fruits characterised by a wide variety of flavours, and differing in texture, skin colour and other sensorial and nutritional parameters. They are usually either white or yellow-fleshed, melting or non-melting, freestone or clingstone, and low-acid or high-acid in flavour. Another key characteristic is ripening time, which correlates with flavour characteristics dictated by the accumulation of sugars, acidity and production of a wide range of low-molecular weight volatile organic compounds (VOCs) [3].

VOCs are responsible for the unique aromatic profile of each cultivar (e.g., [4]). However, only a limited number of VOCs contribute significantly to the final aroma perception [4], and some are often found in very small amounts, detectable only through high resolution methods. Therefore, it is important to link analyses of VOCs to sensorial analyses of aroma perception. VOCs are mostly synthetized by enzymes as part of primary or secondary metabolism, therefore the genetic background is a key component of aromatic profile variation across cultivars [5].

Post-harvest storage conditions also affect peach aroma. Cold temperatures and the use of controlled atmosphere (CA) are necessary to prevent premature fruit decay [6], especially during storage and transport of peaches to different countries, which can take several days and up to 3 weeks, depending on logistics and markets. However, such conditions can result in physiological damage, called ‘chilling injury’, often associated with texture changes and internal browning [6], that only becomes evident after purchase and exposure to room temperature. Chilled storage, even if it does not result in chilling injury, can affect fruit quality resulting in loss of taste and flavour, and storage conditions such as prolonged refrigeration significantly affect VOC production resulting in a loss of taste and flavour [7]. VOC analysis can provide information on changes in composition and abundance of individual VOCs after harvesting and refrigeration, which result in changes in perceived aroma [4,8].

Major contributors to taste perception are well-balanced sugar and acid composition, as well as VOC levels, all of which depend on both pre- and post-harvest factors, including different treatments, fruit maturation stage and storage conditions [8]. Sugar content is often assessed as Brix, which is a measure of the total soluble solids (TSS), since it is a fast measurement that can be done at any stage of the supply chain. The Brix scale, indicating sugar concentration, is often used in combination with firmness and acidity level, indicated as pH or titratable acidity (TA) for determining stage of maturity [9]. In general, as fruits ripen, sugars are released from starch breakdown while acids are degraded, thus the Brix index increases while TA falls. The Brix value mostly depends on pre-harvest conditions, and sugar levels tend to increase under cold storage conditions [10], although genetic background is also important [11]. Accordingly, significant differences among cultivars have been found in sweetness/acidity ratio, and a higher ratio is associated with better consumer acceptance in nectarines and peaches [12]. 

Sensorial studies are widely used to assess peach characteristics, either using a small number of trained panellists or larger, untrained consumer panels. In addition to perceived sweetness and acidity, other sensorial parameters are often evaluated by trained panellists. Astringency, bitterness, crunchiness, firmness, fruitiness, harmony, and juiciness are often used as descriptors, although the choice varies among studies [7,13]. TA and TSS are reliable predictors of perceived sourness and sweetness, respectively; however, they cannot be used as a substitute for their perceived values during sensory evaluation [13]. Fruit juiciness depends on firmness and can either increase or decrease during shelf-life, depending on the cultivar [14]. Interestingly, in studies where peaches were evaluated directly by consumers, their satisfaction rate depended on perceived sweetness, hardness, juiciness, and flavour, but they were unable to appreciate differences in sourness [15].

Additional components of fruit quality and nutritional value are the total carotenoid and total phenolic content, as well as β-carotene and vitamin C concentrations [12,16]. Phenolic compounds are a family of secondary metabolites showing significant antioxidant properties and acting as free radical scavengers, therefore preventing or delaying the onset of a plethora of degenerative diseases [17]. In peach, the most relevant phenolics are chlorogenic acid and catechins, both found in higher amounts in the skin than in the flesh [18]. High antioxidant activity is also related to hydroxycinnamates like chlorogenic acid and flavan-3-ols, including catechin. Flavonols, mainly cyanidin 3-glucoside, quercetin 3-glucoside and cyanidin- 3-rutinoside, are also found at lower concentrations [18]. The resulting phenolic profile varies significantly among cultivars, and with maturation stage at harvest. Although no general rules can be established, late-harvested peaches and nectarines tend to have a higher antioxidant potential [19]. Flesh pigmentation also appears to play a strong role in phenolic composition [20]. 

Carotenoids also contribute to antioxidant power. The most abundant carotenoids in yellow-fleshed peaches include α- and β-carotene (both vitamin A precursors), cryptoxanthin, lutein, and zeaxanthin, while only traces are found in white-fleshed fruits [21]. In peaches, violaxanthin is the predominant xanthophyll both in the skin and in the flesh. It can be found in amounts up to three times higher than other carotenoids [18]. Interestingly, values are highly influenced by post-harvest treatments including refrigeration, and lower temperatures slow down carotenoid biosynthesis [22]. 

Vitamin C (ascorbic acid and dehydroascorbic acid) is an important bioactive compound that also confers health-promoting properties to fruits, and is found in similar amounts in peaches and nectarines. Higher levels are associated with higher density fruits and postharvest ripening [23]. However, vitamin C concentration was observed to decrease with storage at low temperatures [12]. Despite their relevance to human health, to date relatively few studies have investigated how carotenoids, polyphenols and vitamin C levels are related to ripening stage and cultivar [12,20,21,22].

Among the physical parameters, firmness is checked by measuring resistance to vertical penetration. High firmness values are found in fruits harvested when still unripe and are associated with a reduced flavour perception and juiciness. Conversely, a lower firmness is associated with ripening [9], but an excessive loss of firmness over shelf-life is indicative of decay. Storage temperature affects changes in firmness [24]. In comparative studies, consumers seem to prefer relatively less firm cultivars, showing higher TSS and lower TA values, like ‘Big Top’ [20]. Firmness is also often associated to TA, acidity, and sourness, as well as a better ‘grass flavour’ perception [9].

Thus, sensorial perception, a key factor in consumer satisfaction and repurchasing [25] is influenced by the interactions between the cultivar-specific VOC profile, TSS, sweetness and physical characters such as juiciness and firmness. Non-destructive chemical and physical analyses [26] can be used in combination with sensory evaluation to assess fruit quality and predict consumer acceptance [9]. The response to chilled storage is not uniform across peach and nectarine cultivars [10,27], and an understanding of the correlation between VOC changes and chemical and sensorial qualities after storage would enable selection of the best cultivars for long-distance transport as well as selective breeding to identify cultivars showing more resilience to chilling.

Here, we use a combination of VOC, intrinsic quality character and sensorial analyses over six peach and nectarine cultivars to better understand the relationships among all these factors. We link VOC changes across cultivars and during storage to sensorial perception, identifying differences across cultivars in their resilience to chilled storage.

## 2. Materials and Methods

### 2.1. Plant Material

Fruit material, post-harvest conditions and VOC analysis are as described in [27].

### 2.2. Extraction and Analysis of Carotenoids

The extraction of carotenoids was performed as described previously, with some modifications [18]. Briefly, 2 g of finely ground tissue was placed in a flask with 15 mL of hexane and 10 mL of methanolic KOH (100 g/L). The mixture was stirred overnight at room temperature. After the addition of 10 mL of water, in order to remove lipids and KOH, the sample was centrifuged, and the hexane top layer was transferred to a 50 mL Falcon^TM^ tube. The extract was dried using nitrogen gas flow and re-solubilized in 0.5 mL of ethanol. 

The analyses of carotenoids were performed using a Water FractionLynx System (Mildford, MA, USA) equipped with a quaternary gradient pump (Waters 2535) and a UV/Visible detector (Waters 2767). The analytical column used for all chromatographic separations was a reverse-phase Hypersil Gold C_18_ (250 × 4.6 mm; Thermo Fisher Scientific, Rome, Italy). The elution was carried out using acetonitrile, methanol, dichloromethane, and hexane (55:22:11.5:11.5), with 0.02% ammonium acetate, under isocratic conditions at 1 mL/min flow. The run time was 20 min, with an injection volume of 20 µL and the detector set at 454 nm. β-carotene, β-cryptoxanthin, zeaxanthin and lutein were identified and quantified using pure standards, and their content expressed as mg/kg of fresh mass. Pure standards were purchased from Extrasynthese (Genay Cedex, France), with purity (HPLC) ≥92%. For β-carotene the calibration curve was obtained by injecting the standard solutions at the following concentrations: 1, 1.5, 3, 6 and 12 mg/L; the standard solutions of β-cryptoxanthin ranged from 0.625 to 10 mg/L; for zeaxanthin, the calibration curve was in the range of 1.5 and 10 mg/L; meanwhile, for the lutein, the calibration solutions ranged from 1.25 and 10 mg/L.

### 2.3. Extraction and Determination of Total Phenol Content

Frozen fruit material (5 g) was extracted in 10 mL of H_2_O/MeOH (2:8 *v*/*v*) containing 2 mM NaF, in order to prevent phenol oxidation. The mixture was homogenized with an Ultra-Turrax and centrifuged at 6000 rpm for 10 min. The supernatant was filtered with a 0.45 µm PTFE filter and used for the determination of total phenolic compounds [28]. 

A Folin–Ciocalteu assay was performed to evaluate the total content of phenols, and 100 µL of extract was mixed with 100 µL of Folin–Ciocolteu’s reagent. Sodium carbonate (1 mL of 75 g/L) was added, and the resulting mixture was diluted to 5 ml with water. The mixture was kept in the dark at room temperature for 1 h. After this time, the absorbance was measured at 765 nm by using a UV/Vis Spectrophotometer Cary 50 Scan (Varian Inc, Palo Alto, CA, USA). Gallic acid was used as a standard to build the calibration curve, which was prepared using solutions at concentrations from 10 to 100 mg/L. The total phenolic compound content was expressed as mg of gallic acid equivalent per 100 g of fresh pulp. All measurements were carried out in triplicate.

### 2.4. Extraction and Analysis of Vitamin C

For the determination of vitamin C, each peach sample was extracted according to [29] with a few modifications. Frozen pulp (2 g) was mixed with 2 mL of 0.1% formic acid in order to avoid L-ascorbic acid oxidation. The mixture was homogenized for 3 min by vortexing, and then centrifuged at 12,000 rpm for 3 min. The resulting supernatant was diluted to 5 mL using 0.1% formic acid and analysed by HPLC-UV using the same system as above for carotenoids. The column used for the chromatographic separation was a Hypersil Gold C18 column (250 × 4.6 mm; Thermo Fisher Scientific). The elution was performed using linear gradients with 0.1% formic acid in water (solvent A) and acetonitrile (solvent B). The gradient steps were the following: 0–10 min, 100 to 80% A, 10–12 min, 80% A isocratic; 12–15 min, 80 to 20% A; 15–17 min, 20% A isocratic; 17–20, 20 to 100% A and 20–30 min, 100% A isocratic to equilibrate the system before starting the new analysis. The total run time was 30 min, the flow rate was 1 mL/min, the UV detector was set at 265 nm and the injection volume was 20 μL. Vitamin C was quantified using an external calibration standard of L-ascorbic acid ranging 2.5 to 20 μg/mL, and expressed as mg/Kg of fresh pulp.

### 2.5. Total Sugar Analysis

About 1 g of peach pulp was homogenized in 3 mL of distilled water and centrifuged at 3000× *g* (ALC centrifuge-model PK130R) for 15 min at room temperature (RT). Anthrone (0.2 g) was dissolved in 100 mL of H_2_SO_4_ and shaken for 30–40 min. One mL of the peach pulp extract was added to 5 mL of anthrone solution, cooled in ice for 5 min and mixed thoroughly. Samples were incubated at 95 °C for 5 min and then cooled on ice. Absorbance of mixture was measured spectrophotometrically at 620 nm and sugar concentrations were calculated using a glucose calibration curve and expressed as mg/g FW.

### 2.6. Qualitative Analysis

Five fruits of each genotype at each time point were chosen at random, and their flesh firmness, soluble solids content (SSC) and Titratable Acidity (TA) were measured. Flesh firmness was assessed on two sides of fruits, sampled after the removal of a 1 mm thick slice of skin, using an FT70 instrument (Wagner, Greenwich, CT, USA) with an 8-mm cylindrical plunger and was expressed as kg/cm^−2^. Soluble solids content was measured with an optical refractometer MA871 (Milwaukee, Rocky 172 Mount, NC, USA) at the same sites as flesh firmness and was expressed as °Brix. The method for analysis of TA was based on neutralisation of the acids present in the fruit-juice with 0.1 mol/L NaOH. TA values are expressed as percentage of malic acid g/L.

### 2.7. Sensory Evaluation

Two-cm-thick slices were cut for each sample, and each slice was cut in four portions of similar size and placed in an individual plastic dish coded with a three-digit random number. Samples were assessed by a trained panel composed of up to eight panellists selected and trained according to ISO regulations (ISO 8586:2012 Sensory analysis—General guidelines for the selection, training, and monitoring of selected and expert sensory assessors) and having extensive experience in descriptive and quantitative sensory analyses. The descriptors were previously chosen through open discussion between the panel members after tasting different commercial peach and nectarine samples (Table S5). 

Individual samples were served in a randomized order, as established by the Smart Sensory Box program (Smart Sensory Solutions S.r.l., Sassari. Italy). Panellists were seated in isolated, temperature-controlled, and white-lit sensory booths. In each one-hour session, each panellist received a total of eight samples for assessment, served in sequential monadic fashion. Panellists recorded attributes via an iPad logged into a Smart Sensory Box, on a non-structured 10 cm lineal scale, in which 0 meant low intensity of the descriptor and 10 meant high intensity of the descriptor. Water and unsalted crackers were provided for palate cleansing between samples.

The descriptive analysis data were exported from the Smart Sensory Box. Sample means for each of the four biological replications of each genotype were submitted to one-way analysis of variance using Tukey’s test to compare between cultivar after storage, for the different attributes (*p* ≤ 0.05).

### 2.8. Statistical Approaches

All statistical analyses were performed in Rv4.1.1 [30] using RStudio [31]. The effect of cold storage on intrinsic quality characters and on sensory descriptors was modelled using multivariate linear models in mvabund [32]. Significance testing was based on likelihood ratio tests, with *p*-values adjusted for multiple testing via a step-down resampling procedure. Random Forest was used to assess the discriminatory capacity of intrinsic quality characters and sensory descriptors in differentiating between varieties and cold storage treatments, using the package randomForest [33]. Weighted correlation network analysis (WCNA) was used to correlate sensory descriptors with the VOCs and the intrinsic quality characters with a soft-power setting of 5 using ‘flashClust’ and ‘WGCNA’ in R software v.3.6.2 [34].

## 3. Results

### 3.1. Intrinsic Quality Characters among Cultivars before and after Cold Storage

Ten intrinsic quality characters: °Brix, acidity, firmness quality, β-carotene, β-criptoxanthin, zeaxanthin/lutein, total carotenoids, total phenols, vitamin C and total sugars were analysed in three peach cultivars: ‘Rome Star’, ‘Sagittaria’ and ‘Summer Rich’, and three nectarines: ‘Big Bang’, ‘Big Top’ and ‘Carene’ before and after 7 days of storage at 1 °C to assess changes related to cultivar and cold treatment (Table 1). There was a significant overall effect of both cultivar (manylm, F_5,30_ = 133.7, *p* = 0.002) and cold treatment (manylm, F_1,29_ = 124.3, *p* = 0.002); however, the interaction could not be tested without violating model assumptions. Among the individual characters, all except zeaxanthin/lutein and total sugars were significantly affected by cultivar, while acidity, firmness quality, total phenols and vitamin C were significantly affected by cold treatment (Table 2).

**Figure 1:**
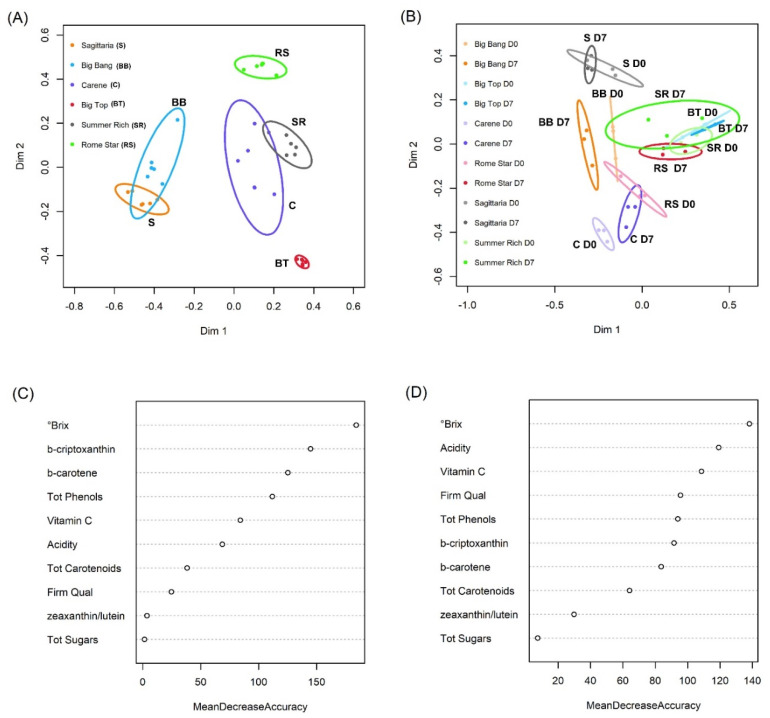
Results from Random Forest cultivar classification based on intrinsic quality characters. (**A**) Multi-dimensional scaling (MDS) plot based on the proximity matrix when D0 and D7 time points are analysed together. (**B**) MDS plot based on the proximity matrix with D0 and D7 time points separated. Each ellipse represents the 95% confidence interval. (**C**) Mean Decrease Accuracy analysis for the Random Forest in panel A. (**D**) Mean Decrease Accuracy analysis for the random forest in panel B. Features are ranked by their contributions to classification accuracy.

Random Forest classification (RF) could differentiate cultivars exceptionally well based on intrinsic quality characters with an out of bag (OOB) error of 2.8%, resulting from a single ‘Sagittaria’ sample misclassified as ‘Big Bang’. Linear discriminant plots showed a clear separation of ‘Rome Star’ and ‘Big Top’ from all other cultivars, whereas ‘Big Bang’ was not discriminated from ‘Sagittaria’, and ‘Summer Rich’ was not discriminated from ‘Carene’; Figure 1A). RF was slightly less successful at distinguishing cultivar and cold treatment together, with an overall OOB error rate of 16.7%. A multi-dimensional scaling (MDS) plot shows separation of most cultivars by storage time except ‘Sagittaria’, and ‘Summer Rich’ (Figure 1B; Table S1). In both cases, °Brix was the most discriminatory parameter, while zeaxanthin/lutein and total sugars were the least informative (Figure 1C,D).

### 3.2. Sensory Descriptors among Cultivars before and after Storage

**Figure 2:**
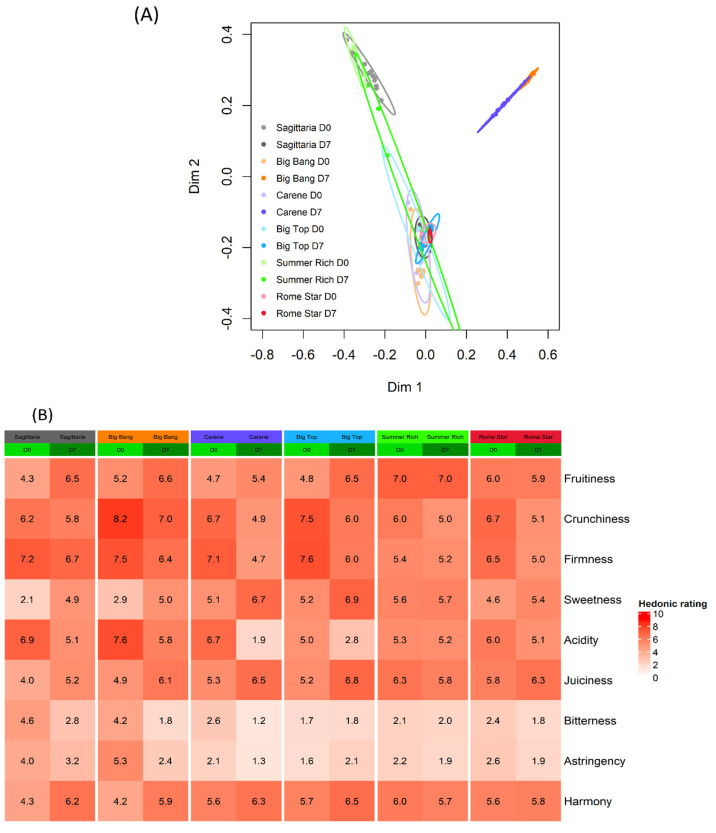
Sensory descriptor variation among cultivars. Fruitiness, crunchiness, firmness, sweetness, acidity, juiciness, bitterness, astringency and harmony were evaluated by a team of trained panellists before (D0) and after (D7) storage; storage was for 7 days at 1 °C followed by a 36 h recovery at 20 °C. Cultivars are shown in order of ripening. (**A**) MDS based on the Random Forest cultivar classification proximity matrix with D0 and D7 time points separated. Each ellipse represents the 95% confidence interval. (**B**) Heatmap of the mean value of each sensory descriptor for each cultivar/storage combination.

A team of 15 trained panellists evaluated all of the peach and nectarine cultivars sampled at D0 and D7. Sensorial analysis was carried out using a hedonic scale (from 0 to 9) of perceived fruitiness, crunchiness, firmness, acidity, juiciness, sweetness, bitterness, astringency, and harmony (Figure 2).

There was a significant interaction overall between the effects of cultivar and cold treatment (*manylm*, F_5,87_ = 57.0, *p* = 0.002) as well as for all of the individual characters except crunchiness and juiciness, both of which showed significant effects of cultivar and cold treatment separately (Table 3).

RF based on sensorial characters was much less accurate than RF based on intrinsic quality characters, although some discrimination was still possible. The OOB error rate was 35.4% for cultivar alone and 50.5% for cultivar and cold treatment combined. An MDS plot did not show discrimination among cultivars (Figure S1, Table S2). When cultivars were separated by treatment, an MDS plot showed that after storage ‘Big Bang’ was not discriminated sensorially from ‘Carene’ but was well-separated from all of the other samples. Samples from day 0 and day 7 from three of the cultivars: ‘Sagittaria’, ‘Big Bang’ and ‘Carene’, were well discriminated, while for cv.s ‘Summer Rich’, ‘Rome Star’ and ‘Big Top’ there was no overall sensorial difference due to storage (Figure 2A, Table S3). 

Fruitiness values either remained the same or increased during cold storage, particularly in ‘Sagittaria’, ‘Big Bang’, and ‘Big Top’. No change was observed in ‘Rome Star’ and in ‘Summer Rich’, which after storage was the fruitiest cultivar. The crunchiest cultivar was ‘Big Bang’, and crunchiness consistently decreased with cold storage. Firmness either remained the same or decreased with cold storage. None of the cultivars had a very high perceived sweetness, but sweetness increased, and acidity decreased in all cases except ‘Summer Rich’, where they remained constant. ‘Big Bang’ was one of the two least sweet cultivars and the most acidic at both D0 and D7. Juiciness increased from D0 to D7, apart from a slight decrease in ‘Summer Rich’. Bitterness stayed constant or decreased in all cases. The most bitter variety at D0 was ‘Big Bang’, but it was among the least bitter at D7. Astringency also either decreased with time of storage or remained the same. Harmony tended to increase, and by D7 was very similar among cultivars. The mean for each cultivar-storage combination showed a moderate correlation between perceived and measured firmness (0.68, Pearson’s *t* = 2.96, *p* = 0.014) and acidity (0.61, Pearson’s *t* = 2.43, *p* = 0.035).

### 3.3. VOCs and Intrinsic Quality Characters in Relation to Sensory Parameters

**Figure 3:**
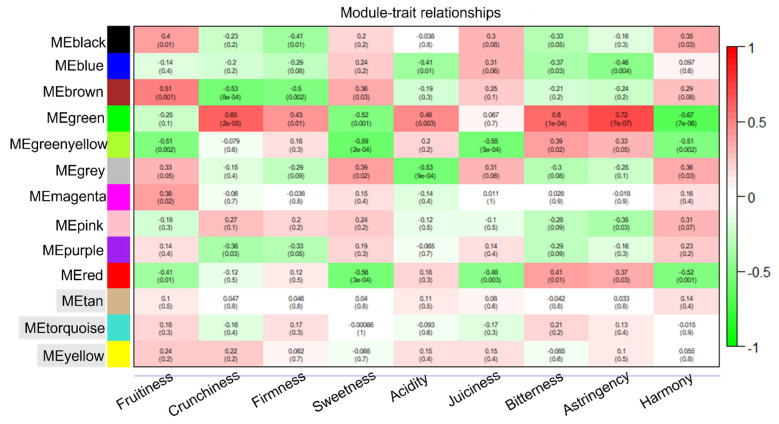
WCNA (Weighted Correlation Network Analysis) showing module–trait relationships. Pearson coefficient and *p*-value (in brackets) are reported. Module names shaded grey indicate lack of significance across all traits.

The ten intrinsic quality parameters described in Section 3.1. were combined with 115 VOCs from the same samples (previously analysed by [27]) in a meta-analysis (Table S6). Weighted correlation network analysis (WCNA) was performed to find relationships between the sensory parameters and the combined VOCs/intrinsic quality character dataset. WCNA uses hierarchical clustering to group compounds into ‘modules’, which are designated arbitrary colour names to distinguish them. For this dataset, WCNA clustered the features into 13 modules (designated by colour names in Figure 3), of which ten showed significant correlation with at least one of the sensory traits. Four of the modules correlated significantly with >4 sensory parameters: seven for the ‘green’ module, six for ‘red’, five for ‘greenyellow’ and four for ‘brown’ (Figure 3 and Table S4). 

**Figure 4:**
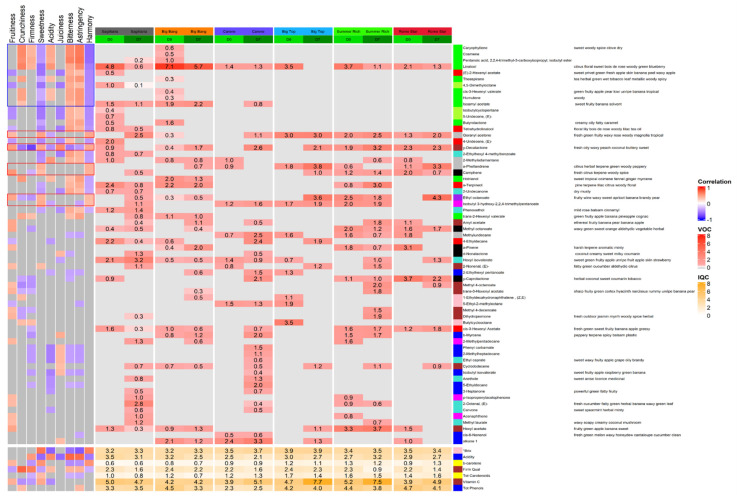
VOCs (**top**) and intrinsic quality parameters (**below**) significantly associated with at least one sensorial attribute, based on WCNA. Grey boxes in the main heatmap indicate that a compound was not detected in that cultivar/day combination. On the left heatmap, grey boxes indicate that a compound was not significantly correlated with that sensorial characteristic; the direction and strength of significant correlations are indicated by the coloured boxes. For VOCs, notes on odour are indicated to the right of each name. Numbers indicate square-root transformed mean relative abundances. Cultivars are shown in order of ripening.Blue box: top 10 VOCs negatively associated with harmony; red boxes: VOCs positively associated with harmony.

The association between sensory parameters and individual VOCs/intrinsic quality characters was investigated (Figure 4): 64 VOCs and seven intrinsic characters significantly associated with at least one sensorial character. Of these, 55 and five, respectively, were also included in modules with an overall significant correlation to a sensorial character. A total of 21 of these VOCs were negatively associated with harmony, a sensorial descriptor which encompasses the overall liking of the fruit. Of the top ten VOCs most negatively associated with harmony, eight were members of the green module. These green module VOCs were also negatively correlated with sweetness and positively correlated with crunchiness, firmness (apart from theaspirane), acidity (except isoamyl acetate), bitterness and astringency. The other two VOCs among the top 10 negatively correlating with harmony were putatively identified as (E)-2-hexenyl acetate from the red module, and 4,5-dimethyloctane from the greenyellow module both of which, unlike the green module VOCs, were also negatively correlated with juiciness and fruitiness, but not positively correlated with acidity, firmness, and crunchiness. Only six VOCs were positively associated with harmony. These were not consistently associated with a single WCNA module, but all showed a positive correlation with sweetness. The majority (4/6) also showed positive correlation with fruitiness and half were negatively correlated with firmness or acidity, or in one case (γ decalactone) both.

Regarding the rest of the significant VOCs and intrinsic quality characters, the majority of the compounds belonging to the red and yellowgreen module shared a similar trend to the green module, with most of them being also negatively correlated with harmony and sweetness. However, unlike those in the green module, these VOCs were also mostly negatively correlated with fruitiness and juiciness. Like the green module, VOCs in this module were also mostly positively correlated with astringency and bitterness. Only one of the VOCs grouped into the brown module showed a significant correlation with harmony: γ decalactone. Most of the VOCs grouping into the brown module were positively correlated with fruitiness and negatively correlated with crunchiness. This module also included VOCs that correlated positively with sweetness and negatively with firmness. The intrinsic characters of firmness quality and Vitamin C also grouped into the brown module. The blue module included total phenolics and acidity as intrinsic characters; this module correlated significantly and negatively with acidity, bitterness, astringency, and firmness while it correlated positively with juiciness (Figure 3 and Figure 4). The remaining intrinsic character that grouped into a module significantly correlating with a sensorial character was Brix, grouped in the pink module. This module was only significantly correlated with astringency, although Brix was positively correlated with harmony and sweetness and negatively correlated with acidity, bitterness, and astringency. Total carotenoids and β-carotene were not grouped into any of the modules showing a significant correlation with a sensorial character, but both of these characters correlated negatively with firmness, bitterness and astringency, and positively with sweetness. β-carotene was also positively correlated with harmony and fruitiness.

### 3.4. VOCs and Intrinsic Quality Characters in Relation to Cultivar and Cold Treatment

The heatmap shown in Figure 4 details the differences in relative abundance in relation to cultivar and cold treatment of all 71 VOCs and intrinsic quality characters that associated significantly with at least one sensorial attribute. The most notable high relative abundance levels were for linalool at day 0 in cv. ‘Big Bang’ which fell with storage, and vitamin C at day 7 in ‘Big Top’ and ‘Summer Rich’, which increased in relation to day 0 of storage.

All of the 21 VOCs that were negatively associated with harmony were entirely absent from ‘Big Top’ and 18 were also absent from the volatilomes of ‘Summer Rich’ and ‘Rome Star’. Of the remaining three VOCs, linalool was detected at both time points of cv.s ‘Summer Rich’ and ‘Rome Star’, 2-methyladamantane at day 7 of ‘Summer Rich’ and day 0 of ‘Rome Star’, and α-terpineol was detected at both day 0 and day 7 in ‘Summer Rich’. ‘Carene’ also emitted only three of these VOCs at day 0: linalool, 2-methyladamantane and phenoxethol, and only two by day 7: linalool and isoamyl acetate. None of the VOCs negatively associated with harmony were consistently present in the other three cultivars at both storage timepoints. The ‘Sagittaria’ volatilome at day 0 had the greatest number of these VOCs (14/21) decreasing to nine at day 7. For ‘Big Bang’ as well the abundance of VOCs negatively regulated with harmony decreased between day 0 and day 7 from twelve to four. The two intrinsic characters negatively correlated with harmony: acidity and firmness quality decreased between day 0 and day 7 in all cultivars except acidity in ‘Summer Rich’ (Figure 4).

Overall, cv.s ‘Big Top’, ‘Summer Rich’ and ‘Rome Star’ included the highest numbers of VOCs associated positively with harmony in their volatilomes, with all six found at day 0 in ‘Summer Rich’. In contrast, five of the six VOCs positively associated with harmony were all absent from the day 0 volatilome of cv. ‘Sagittaria’; the exception was γ decalactone. The day 0 ‘Carene’ volatilome was also low in these VOCs with only two out of the six present (isobutyl 3-hydroxy-2,2,4-trimethylpentanoate and α-phellandrene). In four of the cultivars (‘Sagittaria’, ‘Carene’, ‘Big Top’ and ‘Rome Star’) the number of VOCs linked positively to harmony increased with storage time. Only in ‘Rome Star’ they decreased and in ‘Big Bang’ the number remained stable. Brix and β carotene, both also associated with positive harmony, remained relatively constant between day 0 and day 7 and across all of the cultivars (Figure 4). 

## 4. Discussion

Differences across varieties [4,8] and the effects of chilled storage [10,27] on intrinsic qualities such as sugars and acidity, total phenolic content, and carotenoids, as well as the levels of antioxidant compounds like β-carotene and vitamin C, have been widely documented in peaches and nectarines. Similarly, sensorial perception has been investigated through consumer or trained panel evaluation [9]. Bringing together sensorial and intrinsic quality parameters to assess differences across varieties and differences in their responses to storage is of use in selection of varieties, but few studies have taken this comprehensive approach [7,23], and studies also including VOCs as a part of the same analysis are even fewer [12]. Quality parameters determined by analytical methods represent an objective evaluation; however, there should be a meaningful correlation with the quality attributes appreciated by consumers. Moreover, although some cv.s such as ‘Big Top’ have been studied intensively (e.g., [8,20]), the other cv.s analysed here have received less attention (e.g., [13]). Here, we reveal some interesting correlations among sensorial, intrinsic and VOC characters allowing an assessment of varietal differences both before and after a realistic chilled storage period.

Based only on the intrinsic characters there was some discrimination of the cultivars, which was clearer than that based on sensorial analysis. Interestingly, discrimination was different to that found when using the VOC profiles of the same six cultivars [27]. Brix was one of the most important characters in cultivar discrimination, which fits with changes in sugars across varieties [9]. Brix and acidity were the two most important characters in the discrimination by sample when storage was also considered, and this is consistent with changes related to ripening even during chilling [6]. 

VOC profiles [27] were the most discriminatory between day 0 and day 7 of storage, only failing to discriminate storage timepoints for one cultivar: ‘Big Bang’. Intrinsic characters were able to discriminate storage times in four out of the six cv.s failing only for ‘Sagittaria’ and ‘Summer Rich’. Least discriminatory were the sensorial panels who were unable to discriminate storage timepoints of ‘Sagittaria’, ‘Rome Star’, and ‘Summer Rich’. The lack of discrimination of ‘Summer Rich’ storage times both by sensorial and intrinsic characters fits with a relative lack of change in sensorial characters such as fruitiness, sweetness, and acidity, and essentially no change in Brix, with a relatively small change in acidity. Indeed, fruitiness as a sensorial character was already high compared to the other cultivars at day 0 in ‘Summer Rich’, and thus changes elicited by ripening may have been less easy to detect. The large differences in some sensorial characters across cultivars before storage and the differences in relative change across cultivars is consistent with an overall interaction between cultivar and storage. This is supported by the differences in VOC changes noted previously for these cultivars [27] and for different cultivars in other studies [3]. The WCNA highlighted the linkage among traits related to storage (Figure 3). For example, the green module that was positively correlated with crunchiness was also positively correlated with acidity, bitterness and astringency, and was negatively correlated with sweetness. Conversely the brown module that was positively correlated with fruitiness also correlated positively with sweetness and negatively with crunchiness and firmness.

Of particular interest is the association of aroma compounds with sensorial assessment and their change over storage. Linalool has been previously identified as an abundant VOC in peach skin and flesh [35], showing a particularly low odour threshold. It was found to be essential for perception of the typical ‘fruity’, ‘floral’ flavour in peaches [35] and Chinese raspberry (*Rubus coreanus*; [36]). Although abundant here especially in ‘Big Bang’ before storage, it was negatively correlated with harmony and positively with unripe-related characters. Three other terpenes, caryophyllene, cosmene, and humulene, were also grouped with linalool and were only detected in ‘Big Bang’ at day 0. Some of these terpenes are described as having green, woody aromas. Caryophyllene is very common in the plant kingdom, being responsible for a typical woody, peppery flavour, and is usually found in essential oils of fruit, including others belonging to the genus Prunus (plums) [37]. Cosmene was also found in high amounts in Chinese raspberry and increased with ripening, probably contributing to the final fruity flavour [36]; however, no significant correlations with fruitiness were found in the present study and in ‘Big Bang’ no cosmene was detected after storage when fruitiness was higher in this cultivar. Other terpenes such as α-terpeniol showed a negative correlation to harmony and were relatively highly abundant in ‘Big Bang’ and ‘Sagittaria’ at day 0, that scored poorly on harmony; however, they were also abundant in ‘Summer Rich’ at day 7 which had a better harmony score. Yet other terpenes, such as carvone, dihydrojasmone and β-myrcene, were correlated neither positively nor negatively with harmony. This emphasises the different roles of individual VOCs in the overall aroma bouquet and in different fruit, and may also reflect very different odour thresholds across different terpenes [27,35]. Together with the high crunchiness, firmness and acidity, and low sweetness of ‘Big Bang’ at day 0, these terpenes may contribute to the lack of harmony found for this cultivar pre-storage. In contrast, camphene had a positive correlation to ripening-associated characters. This VOC was absent from the aroma of ‘Big Bang’ but present in the VOC profile of ‘Sagittaria’ and ‘Big Top’ at day 7 and in two other cv.s. It has previously been found to increase during ripening in a raspberry cv. [38], confirming its association with ripening at least in some fruits.

γ- decalactone has also previously been found in peach aroma [35], having a fruity, creamy, peach and apricot-like flavour, and it has been found to be a key peach aroma compound [23]. Here, it was positively associated with harmony and ripening-related sensorial characters, but varied both among cultivars and in response to the storage treatment. Its rise in ‘Big Bang, ‘Carene’, and ‘Big Top’ may contribute to their increased perceived fruitiness and harmony after storage. However, ‘Summer Rich’ actually lost harmony with storage. This cv. was already rated as highly fruity at day 0, so maybe the rise in γ- decalactone was not a major contributor to this sensorial perception.

Esters are another important VOC family contributing to the aroma profile, among which a green leaf volatile, (E)-2-hexenyl acetate, commonly found in peaches [4,35] was also negatively correlated with perceived fruitiness, sweetness and harmony, while being positively correlated with bitterness and astringency. This is perhaps surprising, given that this compound is responsible for a sweet odour and a fresh flavour, with a waxy, apple background. Again, this demonstrates how the contribution of individual VOCs to flavour or taste is context-dependent. The compound was only detected in ‘Sagittaria’ at day 0, which was the least sweet cv. at day 0 and, together with ‘Big Bang’, showed the lowest harmony value, suggesting a possible role for the VOC in the perception of both these sensorial parameters. 

Of the seven intrinsic characters that showed correlation with at least one sensorial character, only Brix and β-carotene showed a positive correlation with harmony. Brix also correlated positively with sweetness and mirrored sweetness ratings, which is consistent with its widespread use as a measure of sugar content in fruit [15,20]. Brix was particularly high in ‘Big Top’ at both day 0 and day 7, and was also high in ‘Carene’ at day 7; both cultivars were also the sweetest, particularly at day 7. Brix values were in line with previous findings [8,15,20]. However, contrary to previous studies [8,12]. Brix levels did not vary significantly during storage. Unexpectedly, total sugar levels were not a highly discriminatory character across cultivars and were not even significantly related to perceived sweetness (*p* > 0.05), which increased in all of the cultivars during storage, albeit to different extents. Although some studies have shown that sweetness significantly decreases during storage, but not °Brix [14], others have found that sweetness increases, together with Brix, as a consequence of flesh dehydration in stone fruits [39]. Thus, our results confirm data from other studies indicating that the overall flavour results from a combination of factors rather than sugar levels alone [9].

Firmness quality in the cultivars was comparable to previous findings [20,26]. Measured firmness fell with storage, and was positively related with perceived firmness, acidity, and sourness in the sensorial analysis, as previously noted [9]. These findings are in agreement with previous studies, showing that measured firmness was generally maintained at 1 °C and 4 °C for the first two weeks of storage, but decreased progressively over time [19,26]. However, the longer the samples were stored under these conditions, the greater the loss of firmness after recovery at ambient temperatures [12]. Interestingly, perceived firmness varied considerably across cultivars both at each timepoint and for each cultivar between timepoints whereas differences in measured firmness were less pronounced. Our findings suggest a subtle difference between measured firmness and sensorial firmness. 

β-carotene was also among the top three intrinsic characteristics able to be discriminated across cultivars in the Random Forest analysis, but was not as important when both cultivar and storage were considered. As shown previously [12], both carotenoids and β-carotene remained relatively constant during storage in all of the cultivars tested. However, all carotenoids were previously found to increase after ripening, in peaches and other crops [12]. This suggests that although ripening continues during storage, some metabolic changes are affected by the cold. Acidity as an intrinsic characteristic correlated well with perceived acidity, as found previously [9], as well as other unripe characters such as firmness and astringency, and negatively with ripening-related characters such as sweetness. Vitamin C levels were in agreement with previous findings [12,20]. Vitamin C levels in four cultivars, ‘Carene’, ‘Big Top’ and ‘Summer Rich’ and ‘Rome Star’, increased with storage, and across the cultivars; vitamin C correlated with sensorial characters fruitiness and sweetness. Previous studies have also shown increasing vitamin C levels over 10 days storage at 6 °C and 4 °C or 20 days at 0 °C [40]. Here, vitamin C levels showed a negative correlation with firmness, while Serra et al. (2020) found a positive correlation. These differences across studies suggest that variety and storage conditions are critical to the vitamin C content available to the consumer [20].

Total phenolics were a useful discriminator among cultivars and days of storage, with levels falling in some cultivars after storage while remaining stable in others. Our findings are therefore in agreement with previous studies reporting no change after harvesting [12] or marked decreases [21]. Although here, varietal differences in phenolics were not as dramatic as previously reported [20], there was nonetheless an over two fold difference at day 0 between ‘Carene’ and all of the other cultivars.

Overall, the combination of the three approaches enabled a link to be made between sensorial assessment and nutritional value. Sensorial traits, like fruitiness and sweetness, are highly associated with better consumer acceptance [25], and here we are able to correlate them with the content of nutritionally relevant compounds. For example, ‘Summer Rich’ was characterized by a particularly high profile in nutritionally relevant compounds: it was very high in vitamin C after storage, and maintained good levels of beta-carotene and total carotenoids. Sensorially, its profile was the fruitiest, with medium-high sweetness, although its acidity was among the highest at day 7. However, it was also characterised by low firmness, making it potentially more sensitive to spoilage. ‘Big Top’ also had a good profile for nutritional content, being high in the antioxidants beta-carotene and total carotenoids, total phenols, and vitamin C. Moreover, ‘Big Top’ was the sweetest cultivar, had low acidity, and was among the fruitiest, juiciest and crunchiest after seven days of storage. This is consistent with data from Serra et al. (2020) showing that sweetness was strongly and positively correlated with both aroma and juiciness. Indeed, confirming previous work [8], firmness was still high after storage. These results are consistent with the popularity of ‘Big Top’, which is one of the most widely grown commercial cultivars at a global level. Previous studies have also indicated that, for this cultivar, consumer satisfaction increases with ripeness and storage temperature as a result of higher perceived sweetness and °Brix values. Sensorial acceptance also remained high after medium-long storage periods [15], and in another study in which trained panellists performed sensorial analysis, ‘Big Top’ was the preferred cultivar, being characterised by an elevated sweetness and juiciness and a relatively low firmness [20]. ‘Big Bang’ had the lowest score with respect to harmony at harvest. Indeed, intrinsic qualities of ‘Big Bang’ were also low, except firmness, which was medium-high. Although it ranked highest at day 7 for crunchiness and had high astringency and acidity, it was also among the fruitiest and juiciest cultivars, and harmony was mid-ranking by day 7. However, it had the lowest vitamin C and the second lowest total phenolic levels after storage. Likewise, ‘Carene’ had low total phenols, along with acidity and total sugars, and all of the other parameters scored low, except °Brix, which was medium-high. In the sensorial analysis, ‘Carene’ had a medium harmony score at harvest but, like ‘Big Bang’, harmony rose considerably during storage to second top place. Although not particularly rich in nutrients, the cultivar can therefore be considered well-balanced.

## 5. Conclusions

The combination of the three approaches into “fruitomics” highlights the importance of sensorial analyses alongside more objective measurements to fully understand varietal characteristics. It also confirms the importance of considering with caution the individual aroma characteristics when evaluating aroma. The correlations between sensorial and intrinsic measurements also reveal important differences, e.g., in the assessment of firmness. Finally, differences revealed in varietal response to storage, in terms both of sensorial appreciation but also nutritional content, are a good starting point for selection of new varieties that combine optimal storage for reduced damage and waste in the supply chain, with maintenance of nutritional value and sensorial appreciation. 

## Figures and Tables

**Table 1:** Peach intrinsic quality characters broken down by cultivar and storage treatment.

Cultivar	Day	Acidity	β Carotene	β Cryptoxanthin	Soluble Solids Content (SSC)	Firmness Quality	Total Carotenoids	Total Phenols	Total Sugars	Vitamin C	Zeaxanthin/ Lutein
		% of Malic Acid g/L	mg/Kg FW	mg/Kg FW	°Brix	Kg/cm^−2^	mg/Kg FW	mg/100 g FW	mg/g FW	mg/Kg FW	mg/Kg FW
Sagittaria	D0	12.24 ± 0.27	0.36 ± 0.08	0.14 ± 0.04	10.3 ± 0.12	5.47 ± 0.68	1.09 ± 0.36	10.87 ± 1.09	32.4 ± 12.36	24.54 ± 1.71	0.59 ± 0.27
	D7	9.47 ± 1.04	0.42 ± 0.01	0.10 ± 0.03	10.8 ± 0.00	2.72 ± 0.71	0.73 ± 0.20	12.13 ± 0.63	37.68 ± 14.37	21.88 ± 2.33	0.35 ± 0.12
Big Bang	D0	9.96 ± 0.15	0.59 ± 0.03	0.070 ± 0.004	10.0 ± 0.00	5.77 ± 0.12	1.38 ± 0.29	20.22 ± 0.32	12.22 ± 8.04	17.35 ± 0.55	0.72 ± 0.26
	D7	6.48 ± 0.19	0.50 ± 0.17	0.02 ± 0.03	11.0 ± 0.00	4.82 ± 0.13	0.52 ± 0.21	11.18 ± 1.47	24.66 ± 7.08	17.66 ± 0.36	0.31 ± 0.53
Carene	D0	6.30 ± 0.12	0.76 ± 0.05	0.080 ± 0.003	11.9 ± 0.12	4.66 ± 0.37	1.38 ± 0.20	5.33 ± 0.08	19.31 ± 6.18	15.55 ± 1.70	0.54 ± 0.17
	D7	4.27 ± 0.64	0.77 ± 0.10	0.10 ± 0.01	14.0 ± 0.00	2.62 ± 0.47	1.70 ± 0.18	6.07 ± 0.62	15.47 ± 9.52	25.54 ± 0.48	0.83 ± 0.12
Big Top	D0	9.20 ± 0.27	1.53 ± 0.57	0.15 ± 0.05	15.0 ± 0.00	5.79 ± 0.46	2.84 ± 0.71	17.71 ± 1.71	30.56 ± 16.01	22.04 ± 1.06	1.16 ± 0.23
	D7	7.24 ± 0.12	1.20 ± 0.27	0.14 ± 0.04	14.8 ± 0.29	5.07 ± 0.07	1.89 ± 0.28	16.39 ± 1.49	35.59 ± 4.86	59.41 ± 2.75	0.55 ± 0.08
Summer Rich	D0	7.44 ± 0.70	1.75 ± 0.29	0.13 ± 0.03	11.8 ± 0.29	5.17 ± 0.41	2.66 ± 0.62	19.14 ± 2.88	32.68 ± 7.88	27.16 ± 2.12	0.78 ± 0.33
	D7	10.27 ± 0.39	1.48 ± 0.40	0.10 ± 0.04	12.0 ± 0.00	0.86 ± 0.27	2.45 ± 1.07	14.12 ± 0.31	25.18 ± 16.83	56.23 ± 5.38	0.87 ± 0.75
Rome Star	D0	8.24 ± 0.00	0.89 ± 0.07	0.06 ± 0.01	12.3 ± 0.00	5.01 ± 0.3	1.85 ± 0.46	21.76 ± 2.40	25.13 ± 27.83	15.59 ± 1.04	0.90 ± 0.41
	D7	7.15 ± 0.19	1.73 ± 0.11	0.04 ± 0.02	11.3 ± 0.12	2.12 ± 0.77	2.60 ± 0.33	16.49 ± 1.82	23.08 ± 4.46	23.87 ± 2.05	0.82 ± 0.23

All figures are shown to two decimal places except °Brix, which could only be measure to one decimal place. The mean of three biological replicates ±SD is shown.

**Table 2:** Univariate test results from the *manylm* model regressing cultivar and storage treatment against peach intrinsic quality characters.

	Acidity		β Carotene		β Cryptoxanthin		°Brix		Firmness Quality		Total Carotenoids		Total Phenols		Total Sugars		Vitamin C		Zeaxanthin/ Lutein	
	LR	*p*	LR	*p*	LR	*p*	LR	*p*	LR	*p*	LR	*p*	LR	*p*	LR	*p*	LR	*p*	LR	*p*
**Cultivar**	**10.16**	**0.002**	**22.18**	**0.002**	**7.96**	**0.002**	**46.5**	**0.002**	**2.67**	**0.003**	**11.16**	**0.002**	**23.23**	**0.002**	2.25	0.14	**6.37**	**0.002**	1.27	0.26
**Storage**	**13.56**	**0.013**	0.21	0.842	4.93	0.145	5.75	0.126	**54.04**	**0.002**	2.38	0.316	**12.11**	**0.016**	0.28	0.842	**28.24**	**0.002**	2.85	0.297

Comparisons are based on likelihood ratio tests. *p*-values are adjusted for multiple testing via a step-down resampling procedure. Significant effects are indicated in bold.

**Table 3:** Univariate test results from the *manylm* model regressing cultivar and storage treatment against peach sensorial characteristics.

	*Acidity*		*Astringency*		*Bitterness*		*Crunchiness*		*Firmness*	
	LR	*p*	LR	*p*	LR	*p*	LR	*p*	LR	*p*
*Cultivar*	8.99	0.002	19.74	0.002	9.24	0.002	**11.2**	**0.002**	6.84	0.002
*Storage*	93.14	0.002	24.4	0.002	33.78	0.002	**64.64**	**0.002**	58.29	0.002
*Cultivar-storage*	**12.96**	**0.002**	**14.96**	**0.002**	**6.15**	**0.003**	2.08	0.112	**4.67**	**0.006**
	**Fruitiness**		**Harmony**		**Juiciness**		**Sweetness**			
	**LR**	***p***	**LR**	***p***	**LR**	***p***	**LR**	***p***		
*Cultivar*	4.91	0.002	3.78	0.003	**4.75**	**0.002**	12.69	0.002		
*Storage*	36.22	0.002	31.01	0.002	**27.17**	**0.002**	81.56	0.002		
*Cultivar-storage*	**4.28**	**0.009**	**4.97**	**0.006**	2.26	0.112	**4.71**	**0.006**		

Comparisons are based on likelihood ratio tests. *p*-values are adjusted for multiple testing via a step-down resampling procedure. Significant effects are indicated in bold (main effects are not shown in bold if involved in a significant interaction).

## Data Availability

Data available upon request.

## Supplementary Materials

The following supporting information can be downloaded at https://www.mdpi.com/article/10.3390/foods11172554/s1, Figure S1: Sensor descriptor variation among cultivars; Table S1: Confusion matrix from a Random Forest classification of peach cultivars and storage treatments based on intrinsic quality characters; Table S2: Confusion matrix from a Random Forest classification of peach cultivars based on sensorial descriptors.; Table S3: Confusion matrix from a Random Forest classification of peach cultivars and storage treatments based on sensorial descriptors.; Table S4: Output from Weighted Correlation Network Analysis of VOCs and intrinsic quality characters vs sensorial traits; Table S5: Peach and nectarine sensory attributes and description used for sensory analysis; Table S6: Mean of three biological replicates of the relative abundance of each VOC detected across each cultivar-storage combination, plus/minus standard deviation. Values have been normalised to total area and square rooted.
