# Supplementary material for: Fruitomics: The Importance of Combining Sensory and Chemical Analyses in Assessing Cold Storage Responses of Six Peach (Prunus persica L. Batsch) Cultivars
Source: Foods. 2022 Aug 24;11(17):2554. doi: 10.3390/foods11172554 (PMC9455255; doi:10.3390/foods11172554)
Supplement: Supplementary file 1 [file foods-11-02554-s001.zip › Supplementary docs- FOR SUBMISSION.pdf]

SUPPLEMENTARY MATERIALS

Type of the Paper (Article)

Fruitomics: the importance of combining sensory and chemical analyses in assessing cold storage responses of six peach (*Prunus persica* L. Batsch) cultivars

Antonella Muto[1#], Sarah R. Christofides[2#], Tiziana Maria Sirangelo[1,3], Lucia Bartella[4], Carsten Muller[2], Leonardo Di Donna[4], Innocenzo Muzzalupo[5], Leonardo Bruno[1], Antonio Ferrante[6], Adriana A. C. Chiappetta[1], Maria Beatrice Bitonti[1], Hilary J. Rogers[2*], Natasha Damiana Spadafora[1,7,8]

[1] Department of Biology, Ecology and Earth Sciences, University of Calabria, Via Ponte P. Bucci 6b, 87036 Arcavacata di Rende, Cosenza, Italy
[2] School of Biosciences, Cardiff University, Sir Martin Evans Building, Museum Avenue, Cardiff, United Kingdom
[3] CREA—Council for Agricultural Research and Agricultural Economy Analysis, Genomics and Bioinformatics research center, 26836, Montanaso Lombardo Lodi, Italy
[4] Department of Chemistry and Chemical Technologies, University of Calabria, Via Ponte P. Bucci 15c, 87036 Arcavacata di Rende Cosenza, Italy
[5] CREA—Council for Agricultural Research and Agricultural Economy Analysis, Forestry and Wood research center, 87036 Rende (CS), Italy
[6] Department of Agricultural and Environmental Science – Production, Landscape, Agroenergy, Università degli Studi di Milano, Via Celoria 2, 20133, Milan, Italy
[7] Markes International Ltd, 1000B Central Park, Western Avenue, CF31 3RT, Bridgend, United Kingdom
[8] Department of Chemical, Pharmaceutical and Agricultural Sciences, University of Ferrara, 44121, Ferrara, Italy
* Correspondence: rogershj@cardiff.ac.uk; Tel: +44 (0)2920876352

\# These authors contributed equally to this work.

Table S1. Confusion matrix from a Random Forest™ classification of peach cultivars and storage treatments based on intrinsic quality characters.

		Sagittaria		Big Bang		Carene		Big Top		Summer Rich		Rome Star		% Correct
		D0	D7	D0	D7	D0	D7	D0	D7	D0	D7	D0	D7	
**Sagittaria**	**D0**	2	1	0	0	0	0	0	0	0	0	0	0	67
**Sagittaria**	**D7**	0	3	0	0	0	0	0	0	0	0	0	0	100
**Big Bang**	**D0**	0	0	3	0	0	0	0	0	0	0	0	0	100
**Big Bang**	**D7**	0	0	0	3	0	0	0	0	0	0	0	0	100
**Carene**	**D0**	0	0	0	0	3	0	0	0	0	0	0	0	100
**Carene**	**D7**	0	0	0	0	0	3	0	0	0	0	0	0	100
**Big Top**	**D0**	0	0	0	0	0	0	2	1	0	0	0	0	67
**Big Top**	**D7**	0	0	0	0	0	0	0	3	0	0	0	0	100
**Summer Rich**	**D0**	0	0	0	0	0	0	1	0	2	0	0	0	67
**Summer Rich**	**D7**	0	0	0	0	0	0	0	1	0	2	0	0	67
**Rome Star**	**D0**	0	0	0	1	0	0	0	0	0	0	2	0	67
**Rome Star**	**D7**	0	0	0	0	0	0	0	0	1	0	0	2	67

Rows represent true categories; columns represent predicted categories. The number of replicates correctly predicted for each category is highlighted in green. Incorrectly predicted replicates are highlighted in red. Cultivars are shown in order of ripening. Note that replicate numbers differ between cultivars based on the number of panellists available for each session.

Table S2. Confusion matrix from a Random Forest™ classification of peach cultivars, based on sensorial descriptors.

	Sagittaria	Big Bang	Carene	Big Top	Summer Rich	Rome Star	No. reps	% Correct
**Sagittaria**	15	2	0	1	0	1	19	79
**Big Bang**	1	12	2	2	1	1	19	63
**Carene**	2	0	10	2	0	2	16	63
**Big Top**	0	1	3	15	1	1	21	71
**Summer Rich**	0	0	0	1	8	3	12	67
**Rome Star**	2	1	1	1	3	4	12	33

Rows represent true categories; columns represent predicted categories. The number of replicates correctly predicted for each category is highlighted in green. Incorrectly predicted replicates are highlighted in red. Cultivars are shown in order of ripening from early to late. Note that replicate numbers differ between cultivars based on the number of panellists available for each session.

Table S3. Confusion matrix from a Random Forest™ classification of peach cultivars and storage treatments based on sensorial descriptors.

		Sagittaria		Big Bang		Carene		Big Top		Summer Rich		Rome Star		No. reps	% Correct
		D0	D7	D0	D7	D0	D7	D0	D7	D0	D7	D0	D7		
**Sagittaria**	**D0**	8	0	1	0	0	0	0	0	0	0	0	0	9	89
**Sagittaria**	**D7**	2	5	0	1	0	0	1	0	0	0	1	0	10	50
**Big Bang**	**D0**	2	0	9	0	0	0	0	0	0	0	0	0	11	82
**Big Bang**	**D7**	0	2	0	1	2	0	1	0	0	1	1	0	8	13
**Carene**	**D0**	0	1	0	1	3	0	3	0	0	0	1	0	9	33
**Carene**	**D7**	0	0	0	0	0	6	0	1	0	0	0	0	7	86
**Big Top**	**D0**	0	2	0	0	2	0	6	0	0	0	1	0	11	55
**Big Top**	**D7**	0	0	0	0	0	1	0	9	0	0	0	0	10	90
**Summer Rich**	**D0**	0	0	0	1	0	0	0	0	1	3	1	1	7	14
**Summer Rich**	**D7**	0	0	0	0	0	0	0	0	3	1	0	1	5	20
**Rome Star**	**D0**	1	2	0	1	1	0	1	0	1	0	0	0	7	0
**Rome Star**	**D7**	0	0	0	0	1	0	1	0	0	3	0	0	5	0

Rows represent true categories; columns represent predicted categories. The number of replicates correctly predicted for each category is highlighted in green. Incorrectly predicted replicates are highlighted in red. Cultivars are shown in order of ripening. Note that replicate numbers differ between cultivars based on the number of panellists available for each session.

Table S5: Peach and nectarine sensory attributes and description used for sensory analysis.

Attributes	Description
Fruitiness	Strength of fruity flavour
Crunchiness	Hard texture with a low-pitched sound
Firmness	Peak force required during the first sample compression cycle
Sweetness	Fundamental taste detectable on the tip of the tongue, characteristic of sugars
Acidity	fundamental taste detectable on the sides of the tongue (e.g. citric acid)
Juiciness	Amount of juice released on the first chews of the sample
Bitterness	Characteristic flavor reminding of unripe fruit, more or less pleasant depending on intensity
Astringency	A puckering sensation in the mounth characteristic of tannins
Harmony	Overall liking of the fruit

Table S6. Mean of three biological replicates of the relative abundance of each VOC detected across each cultivar-storage combination, plus/minus standard deviation. Values have been normalised to total area and square rooted.

	Sagittaria		Big Bang		Carene		Big Top		Summer Rich		Rome Star	
	D0	D7	D0	D7	D0	D7	D0	D7	D0	D7	D0	D7
2,4-Dimethyl-1-heptene	-	-	0.21 ± 0.16	-	-	-	-	-	-	-	1.06 ± 0.90	-
Isoamyl acetate	1.53 ± 1.32	1.11 ± 0.95	1.88 ± 1.09	2.21 ± 0.13	-	0.78 ± 0.72	-	-	-	-	-	-
3-Heptanone	-	0.51 ± 0.44	-	-	-	0.72 ± 0.75	-	-	-	-	-	-
trans-1,2-Diethyl cyclopentane	-	-	0.33 ± 0.28	-	-	-	1.51 ± 1.69	-	-	0.76 ± 0.06	0.83 ± 0.81	-
Amyl acetate	-	0.40 ± 0.32	-	1.12 ± 0.29	-	0.50 ± 0.43	-	-	-	1.77 ± 0.54	1.06 ± 0.92	-
cis-6-Nonenol	-	-	-	-	0.52 ± 0.43	0.6 ± 0.71	-	-	-	-	-	-
Methyl caproate	-	-	-	-	-	-	-	-	-	-	-	1.12 ± 0.95
3-Ethyl-2-methylheptane	-	-	-	-	-	-	-	-	0.51 ± 0.42	-	0.75 ± 0.62	0.5 ± 0.41
Butylcyclopentane	-	0.48 ± 0.40	-	-	1.26 ± 1.13	-	-	-	-	-	-	-
4,5-Dimethyloctane	1.01 ± 0.86	0.10 ± 0.13	-	-	-	-	-	-	-	-	-	-
Camphene	-	0.51 ± 0.43	-	-	-	-	-	0.98 ± 0.82	1.17 ± 0.35	1.37 ± 0.27	1.95 ± 0.15	0.69 ± 0.58
3-Octanone	-	-	-	0.50 ± 0.45	-	-	-	-	-	-	-	-
Isovalerone	-	0.33 ± 0.26	-	-	3.08 ± 0.50	-	1.25 ± 1.06	1.81 ± 1.77	1.23 ± 1.62	0.92 ± 0.78	1.91 ± 2.24	1.52 ± 1.57
α-Pinene	-	-	0.36 ± 0.33	1.96 ± 1.80	-	-	-	-	1.76 ± 0.68	0.74 ± 0.62	3.05 ± 0.75	-
Butylcyclooctane	-	-	-	-	-	-	3.52 ± 1.79	-	-	-	-	-
β-Myrcene	-	-	0.82 ± 0.39	1.25 ± 0.86	-	2.00 ± 1.75	-	-	1.54 ± 1.31	1.73 ± 1.48	-	-
3-Methylnonane	-	-	0.61 ± 0.59	1.92 ± 1.67	2.30 ± 3.13	-	2.68 ± 3.26	-	-	1.38 ± 1.20	2.00 ± 1.23	-
3,5-Dimethylheptane	-	-	-	-	-	-	-	-	-	-	1.37 ± 1.37	-
terpene 1	-	-	-	-	0.72 ± 0.60	-	-	-	-	-	-	-
Sulcatol	-	-	-	0.59 ± 0.50	-	-	-	-	-	-	-	-
trans-3-Hexenyl acetate	-	-	-	0.34 ± 0.27	-	-	-	-	-	1.76 ± 0.65	-	-
α-Phellandrene	-	-	-	0.74 ± 0.81	0.92 ± 0.07	-	1.80 ± 1.29	3.81 ± 0.62	0.60 ± 0.65	-	1.10 ± 0.93	3.34 ± 1.44
cis-3-Hexenyl Acetate	1.56 ± 1.22	0.27 ± 0.21	0.95 ± 1.38	0.57 ± 0.63	-	0.69 ± 0.60	-	-	1.61 ± 0.38	1.74 ± 0.57	1.24 ± 1.37	1.78 ± 1.00
3-Hexyne	-	-	-	-	-	-	-	-	0.73 ± 0.63	-	-	-

Phenyl carbamate	-	-	-	-	-	1.46 ± 1.26	-	-	-	-	-	-
Hexyl acetate	1.30 ± 0.63	0.34 ± 0.28	0.92 ± 0.05	1.34 ± 0.91	-	-	-	1.12 ± 1.01	3.33 ± 0.71	3.72 ± 0.15	1.48 ± 1.84	-
2,6-Dimethylnonane	2.24 ± 2.65	2.19 ± 0.34	1.00 ± 1.08	1.12 ± 1.11	2.93 ± 2.55	2.01 ± 1.72	-	-	1.23 ± 1.37	-	-	-
(E)-2-Hexenyl acetate	0.54 ± 0.44	-	-	-	-	-	-	-	-	-	-	-
Isobutyl isovalerate	-	-	-	-	-	0.45 ± 0.38	-	-	-	-	-	-
5-Undecene, (E)-	0.74 ± 0.69	-	-	-	-	-	-	-	-	-	-	-
4-Undecene, (E)-	2.01 ± 2.50	-	-	-	-	-	-	-	-	-	-	-
2-Cyclopropylhexane	-	-	-	-	-	-	-	-	-	-	-	1.01 ± 1.03
p-Cymene	-	0.63 ± 0.53	-	-	-	-	-	-	-	-	0.75 ± 0.63	-
5-Ethyl-2-methyloctane	-	-	-	-	1.45 ± 1.25	1.29 ± 1.15	1.93 ± 0.50	-	-	-	-	-
β-Ocimene	-	-	1.16 ± 1.56	-	0.88 ± 0.80	0.77 ± 0.10	0.77 ± 0.65	-	1.21 ± 1.43	-	-	0.65 ± 0.54
alkane 1	-	-	2.10 ± 1.80	1.18 ± 1.56	2.37 ± 2.29	3.27 ± 0.82	-	1.31 ± 1.17	-	-	1.04 ± 0.88	-
Spiro[4.5]decane	-	-	-	0.51 ± 0.49	0.92 ± 0.96	-	-	-	-	0.63 ± 0.53	-	-
Indene	0.60 ± 0.02	0.59 ± 0.01	-	0.77 ± 0.04	1.09 ± 1.04	0.42 ± 0.34	-	1.46 ± 0.32	-	-	-	-
Isobutylcyclopentane	0.35 ± 0.31	-	-	-	-	-	-	-	-	-	-	-
γ-Caprolactone	0.90 ± 0.76	-	-	-	-	2.13 ± 1.76	-	-	1.09 ± 1.09	0.99 ± 0.26	3.69 ± 1.41	2.18 ± 0.58
Butyrolactone	0.54 ± 0.10	-	1.57 ± 2.67	-	-	-	-	-	-	-	-	-
Tetrahydrolinalool	0.79 ± 0.67	0.51 ± 0.42	-	-	-	-	-	-	-	-	-	-
Linalool	4.75 ± 2.15	0.59 ± 0.50	7.11 ± 1.11	5.73 ± 0.22	1.4 ± 1.20	1.33 ± 0.63	3.45 ± 3.03	-	3.69 ± 1.65	1.12 ± 0.97	2.10 ± 0.33	1.34 ± 1.33
Isoamyl valerianate	-	0.33 ± 0.26	0.48 ± 0.58	0.49 ± 0.47	0.77 ± 0.73	0.78 ± 0.27	-	-	-	-	-	-
2-Octenal, (E)-	-	2.83 ± 1.10	-	-	-	0.39 ± 0.31	-	-	0.89 ± 0.76	0.64 ± 0.53	-	-
Hotrienol	-	-	2.00 ± 0.9	1.26 ± 1.69	-	-	-	-	-	-	-	-
Methyl 4-octenoate	-	-	-	-	-	-	-	-	-	1.99 ± 0.26	-	0.91 ± 0.92
3,7-Dimethyldecane	2.43 ± 3.21	0.50 ± 0.41	-	-	-	1.40 ± 2.01	-	-	-	-	1.43 ± 2.42	-
Methyl octanoate	0.44 ± 0.37	0.51 ± 0.43	-	0.37 ± 0.30	-	-	-	-	2.02 ± 0.20	1.19 ± 0.08	1.56 ± 0.60	1.67 ± 0.59
Allo-Ocimene	0.56 ± 0.46	0.38 ± 0.31	0.46 ± 0.51	0.61 ± 0.17	-	0.44 ± 0.36	1.04 ± 0.88	1.53 ± 0.27	1.08 ± 0.32	-	-	-
Cosmene	-	-	0.46 ± 0.06	-	-	-	-	-	-	-	-	-
5-Ethyldecane	-	-	-	-	-	2.02 ± 2.44	-	-	-	-	-	-
4-Ethyldecane	2.15 ± 1.07	0.42 ± 0.35	0.57 ± 0.61	-	-	2.40 ± 0.43	-	1.89 ± 1.61	-	-	-	-
n-Amyl isovalerate	-	-	0.46 ± 0.75	1.06 ± 1.24	1.73 ± 0.97	0.52 ± 0.43	-	-	-	-	-	-
Methylundecane	-	-	-	-	0.75 ± 0.62	2.46 ± 2.16	1.65 ± 1.79	-	1.57 ± 1.43	0.73 ± 0.61	1.77 ± 2.35	-

L-camphor	-	0.35 ± 0.28	-	-	-	-	-	2.69 ± 3.07	-	-	-	-
Benzyl acetate	-	2.41 ± 0.52	-	-	-	0.77 ± 0.64	-	-	-	0.59 ± 0.49	-	-
(3Z,5E)-1,3,5-Undecatriene	-	-	-	0.32 ± 0.26	-	-	-	-	-	-	-	-
2-Methyladamantane	1.00 ± 0.34	-	0.77 ± 0.65	0.75 ± 0.66	0.99 ± 0.84	-	-	-	-	0.64 ± 0.54	0.76 ± 0.63	-
1-p-Tolylethanone	-	1.05 ± 0.12	-	-	-	-	-	-	-	-	-	-
Dihydrojasmone	-	0.33 ± 0.27	-	-	-	-	-	-	-	1.86 ± 0.50	-	-
Ethyl octanoate	-	0.50 ± 0.42	0.27 ± 0.22	0.46 ± 0.37	-	-	-	3.62 ± 1.38	2.54 ± 2.25	1.78 ± 0.91	-	4.25 ± 1.18
2-Nonenal, (E)-	-	1.10 ± 0.01	-	-	0.76 ± 0.64	-	-	1.22 ± 1.03	-	1.49 ± 0.64	-	-
α-Terpineol	2.38 ± 2.49	0.77 ± 0.20	2.25 ± 0.74	2.03 ± 1.84	-	-	-	-	0.77 ± 0.65	2.98 ± 2.58	-	-
2,4-Dimethylundecane	-	-	-	0.40 ± 0.34	-	-	-	-	-	-	-	-
Ethylhexyl 2-propenoate	0.48 ± 0.39	2.42 ± 2.10	-	-	-	-	-	-	-	0.65 ± 0.54	-	-
2,4-Decadienal, (E,E)-	-	0.61 ± 0.20	-	-	-	-	-	-	-	-	-	-
cis-3-Hexenyl valerate	-	-	0.39 ± 0.33	-	-	-	-	-	-	-	-	-
cis-3-Hexenyl isovalerate	0.74 ± 0.23	0.78 ± 0.66	1.29 ± 0.12	2.17 ± 2.06	1.25 ± 1.06	1 ± 0.60	-	-	1.65 ± 0.04	1.78 ± 0.32	-	1.23 ± 1.05
1-Ethyldecahydronaphthalene , (Z,E)	-	-	-	0.45 ± 0.41	-	-	1.09 ± 0.91	-	-	-	-	-
Hexyl isovalerate	2.12 ± 1.77	3.21 ± 2.79	0.47 ± 0.17	0.50 ± 0.11	1.37 ± 1.56	0.88 ± 0.47	0.72 ± 0.70	-	-	0.97 ± 0.83	-	1.31 ± 0.15
trans-2-Hexenyl valerate	-	0.79 ± 0.67	1.12 ± 0.63	1.04 ± 0.87	-	-	-	-	-	-	-	-
Phenoxethol	1.20 ± 1.01	1.43 ± 1.26	-	-	-	-	-	-	-	-	-	-
Cyclododecene	-	0.72 ± 0.61	0.70 ± 0.89	0.50 ± 0.53	-	0.52 ± 0.49	-	1.15 ± 1.00	-	1.07 ± 0.91	-	0.9 ± 0.86
4-Methyldodecane	-	-	0.32 ± 0.26	-	3.03 ± 2.62	-	0.92 ± 0.78	1.45 ± 1.34	-	2.41 ± 0.35	-	-
Carvone	-	0.56 ± 0.15	-	-	-	0.49 ± 0.46	-	-	-	-	-	-
Amyl caproate	-	-	-	0.61 ± 0.07	-	-	-	-	-	-	-	-
Anethole	-	0.82 ± 0.22	-	-	-	1.34 ± 1.44	-	-	-	-	-	-
2-Undecanone	0.68 ± 0.58	0.7 ± 0.59	-	-	-	-	-	-	-	-	-	-
Theaspirane	-	-	0.27 ± 0.20	-	-	-	-	-	-	-	-	-
Methyl 4-decenoate	-	-	-	-	-	-	-	-	-	1.53 ± 0.21	-	-
Nonyl acetate	-	-	-	-	-	-	-	-	-	-	0.82 ± 0.69	0.94 ± 0.91
Bicyclohexane	-	-	0.62 ± 0.35	0.85 ± 0.72	1.31 ± 1.11	0.92 ± 0.77	2.35 ± 1.3	-	1.08 ± 0.57	0.72 ± 0.61	-	0.89 ± 0.74
6-Dodecanone	-	0.47 ± 0.02	-	-	-	-	-	-	-	-	-	-
p-Isopropenylacetophenone	-	1.04 ± 0.37	-	-	-	-	-	-	0.92 ± 0.77	-	-	-
Heptyl isovalerate	-	-	0.65 ± 0.55	0.61 ± 0.12	0.97 ± 0.09	0.87 ± 0.24	-	-	-	-	-	-

Butyl carbitol acetate	-	2.71 ± 0.72	-	-	-	-	-	-	-	-	-	-
Isobutyl 3-hydroxy-2,2,4-trimethylpentanoate	-	1.12 ± 0.99	-	-	1.25 ± 1.30	1.58 ± 0.22	1.73 ± 1.70	1.87 ± 1.67	2.04 ± 1.77	1.95 ± 1.68	-	-
2-Ethylhexyl pentanoate	-	-	-	0.58 ± 0.48	-	1.47 ± 1.37	1.32 ± 1.15	-	-	-	-	-
2-Undecenal	-	1.35 ± 0.14	-	-	-	0.59 ± 0.51	-	-	0.93 ± 0.83	-	-	-
γ-Nonalactone	-	1.30 ± 0.29	-	-	-	0.50 ± 0.41	-	-	-	-	-	-
Ethyl caprate	-	-	-	-	-	0.57 ± 0.49	-	-	-	-	-	-
2-Methyl-1-decanol	-	0.94 ± 0.23	-	-	-	-	-	-	-	-	-	-
Tetrahydrogeranylacetone	-	0.90 ± 0.03	-	-	-	0.43 ± 0.35	-	-	-	-	-	-
Cedr-8-en-15-ol	-	-	-	0.38 ± 0.33	1.45 ± 1.34	-	-	1.18 ± 1.00	1.08 ± 1.21	0.69 ± 0.59	1.85 ± 2.30	1.31 ± 1.39
Isoamyl caprylate	-	-	-	0.94 ± 0.22	-	-	-	-	-	-	-	-
Geranyl acetone	-	2.53 ± 0.93	0.29 ± 0.27	-	-	1.08 ± 0.98	3.00 ± 1.73	3.04 ± 2.80	1.96 ± 1.03	2.46 ± 0.26	1.34 ± 1.41	2.02 ± 0.74
Caryophyllene	-	-	0.58 ± 0.05	-	-	-	-	-	-	-	-	-
Humulene	-	-	0.33 ± 0.27	-	-	-	-	-	-	-	-	-
1-Heptadecanol	-	1.12 ± 0.99	-	0.99 ± 1.67	1.69 ± 1.66	-	1.19 ± 1.03	1.11 ± 1.04	-	-	-	3.71 ± 3.47
γ-Decalactone	0.90 ± 0.16	-	0.38 ± 0.31	1.70 ± 0.49	-	2.55 ± 0.93	-	2.13 ± 0.58	1.90 ± 0.33	3.24 ± 0.30	2.33 ± 0.75	2.30 ± 0.99
Pentyl octanoate	-	-	-	1.05 ± 0.51	-	-	-	-	-	-	-	-
Triethylene dimethacrylate	-	2.34 ± 1.40	-	-	-	-	-	-	-	-	-	-
Acenaphthene	-	1.01 ± 0.41	-	-	-	-	-	-	0.79 ± 0.66	-	-	-
δ-Nonalactone	-	-	-	0.48 ± 0.39	-	0.93 ± 0.18	-	-	-	-	1.18 ± 1.00	-
Methyl laurate	-	1.20 ± 0.32	-	-	-	-	-	-	-	0.72 ± 0.61	-	-
2-Methylpentadecane	-	1.35 ± 1.19	-	0.58 ± 0.58	-	-	-	-	1.58 ± 1.61	-	-	-
Pentanoic acid, 2,2,4-trimethyl-3-carboxyisopropyl, isobutyl ester	-	0.24 ± 0.19	0.98 ± 0.08	-	-	-	-	-	-	-	-	-
2-Tetradecanone	-	0.95 ± 0.07	-	-	-	-	-	-	-	-	-	-
Jasminal	-	0.75 ± 0.09	-	-	-	-	-	-	-	-	-	-
2-Pentadecanone	-	0.69 ± 0.50	-	-	-	-	-	-	-	-	-	-
2-Methylheptadecane	-	-	-	-	-	1.14 ± 0.99	-	-	-	-	-	-
Methyl tetradecanoate	-	1.60 ± 0.70	-	-	-	-	-	-	-	0.72 ± 0.60	-	-
2-Ethylhexyl 4-methylbenzoate	0.76 ± 0.63	0.69 ± 0.17	-	-	-	-	-	-	-	-	-	-
Methyl hexadecanoate	-	2.27 ± 1.52	-	-	-	-	-	-	-	-	-	-

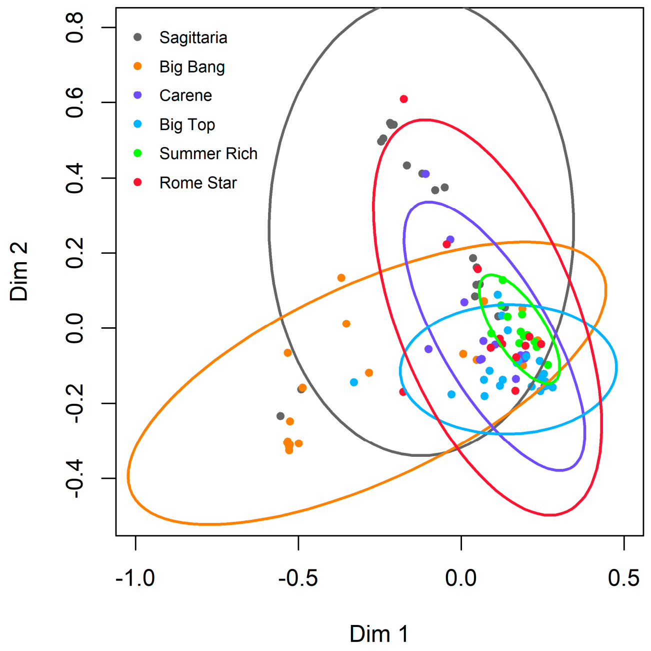

Figure S1. Sensory descriptor variation among cultivars. Fruitiness, Crunchiness, Firmness, Sweetness, Acidity, Juiciness, Bitterness, Astringency and Harmony, were evaluated by a team of trained panellists before (D0) and after (D7) storage; storage was for 7 days at 1 °C followed by a 36 h recovery at 20 °C. Cultivars are shown in order of ripening. Multi dimensional scaling (MDS) plot based on the Random Forest™ cultivar classification proximity matrix when D0 and D7 time points are analysed together.
